# Igualdad de género y equidad en salud: lecciones estratégicas de las experiencias de los países en la incorporación de la perspectiva de género en la salud^*^

**DOI:** 10.26633/RPSP.2021.103

**Published:** 2021-10-18

**Authors:** Ana Cristina González Vélez, Anna Coates, Victoria Diaz Garcia, Denisse Wolfenzon

**Affiliations:** 1 Consultora independiente Bogotá Colombia Consultora independiente, Bogotá, Colombia.; 2 Organización Panamericana de la Salud Washington D.C. Estados Unidos de América Organización Panamericana de la Salud, Washington D.C., Estados Unidos de América.; 3 Dublin City University Dublín Irlanda Dublin City University, Dublín, Irlanda.; 4 Consultora independiente Stamford Estados Unidos de América Consultora independiente, Stamford, Estados Unidos de América.

**Keywords:** Equidad en salud, igualdad de género, transversalidad de género, políticas inclusivas de género, Guatemala, Guyana, Perú, Health equity, gender equality, gender mainstreaming, gender-inclusive policies, Guatemala, Guyana, Peru, Equidade na saúde, igualdade de gênero, transversalidade de gênero, políticas inclusivas de gênero, Guatemala, Guiana, Peru

## Abstract

**Objetivos.:**

Analizar el progreso en las estructuras, mecanismos y estrategias organizativas, así como los factores y las barreras, que favorecen la incorporación de la perspectiva de género en la salud en Guatemala, Guyana y Perú, dado el papel que ello desempeña en el abordaje de las desigualdades de género en la salud como un motor estructural clave de la equidad en salud.

**Métodos.:**

Se obtuvieron datos a partir de la literatura gris de leyes, políticas o documentos de programas y entrevistas cualitativas semiestructuradas con 37 informantes. El análisis se basó en un marco teórico que incluía siete categorías consideradas esenciales para avanzar la incorporación de la perspectiva de género en el sector de la salud.

**Resultados.:**

A pesar de los importantes esfuerzos y las experiencias acumuladas respecto de la incorporación de la perspectiva de género en el sector de la salud persisten obstáculos estructurales, como desafíos sociales más amplios para transformar las relaciones de poder desiguales entre los géneros; la complejidad del sistema de salud combinada con una baja capacidad técnica, política y financiera de las estructuras institucionales encargadas de abordar el tema; y la limitada coordinación con las instituciones nacionales dedicadas a la promoción de la mujer (a menudo, débiles). En algunos contextos, los obstáculos se ven agravados por la limitada comprensión de los conceptos básicos subyacentes a la perspectiva de género (a veces exacerbada por una comprensión limitada de la interseccionalidad o el compromiso con los hombres) y la ausencia de indicadores para medir los resultados y el impacto concreto de la incorporación de la perspectiva de género.

**Conclusiones.:**

Para que la incorporación de la perspectiva de género en la salud sea satisfactoria, se requiere una agenda más estratégica y transformadora, elaborada e implementada en coordinación con las instituciones nacionales de promoción de la mujer y la sociedad civil y vinculada a instancias externas (p. ej., el Comité para la Eliminación de la Discriminación contra la Mujer). Es necesario, asimismo, una distinción más clara entre los enfoques sensibles al género y aquellos transformativos de las relaciones desiguales de género, y una definición de los resultados previstos y los indicadores para medir los avances. Estos podrían entonces documentarse y sistematizarse mejor, lo que permitiría que la perspectiva de género se comprendiera más ampliamente y se pusiera en práctica como instrumento concreto para lograr la equidad en salud.

## Abstract (en)

**Objectives.:**

To analyze progress in organizational structures, mechanisms, strategies, and enabling factors and barriers towards gender mainstreaming (GM) in health in Guatemala, Guyana, and Peru, given GM’s role in addressing gender inequalities in health as a key structural driver of health equity.

**Methods.:**

Data was obtained through a grey literature review of laws, policies, and/or program documents and semi-structured qualitative interviews with 37 informants. Analysis was based on a theoretical framework including 7 categories considered essential to advance GM in the health sector.

**Results.:**

Despite significant efforts and accumulated experiences of GM in health, structural barriers include: wider societal challenges of transforming gender unequal power relations; health system complexity combined with the low technical, political, and financial capacity of institutional structures tasked with GM; and limited coordination with (often weak) National Women’s Machineries (NWMs). In some contexts, barriers are compounded by limited understanding of basic concepts underlying GM (at times exacerbated by misunderstandings related to intersectionality and/or engagement with men) and the absence of indicators to measure GM’s concrete results and impact.

**Conclusions.:**

Successful GM requires a more strategic and transformational agenda, developed and implemented in coordination with NWMs and civil society and with reference to external bodies (e.g. Committee on the Elimination of Discrimination against Women) to go beyond process, with clearer distinction between gender sensitivity and gender transformation, and definition of expected results and indicators to measure advances. These then could be better documented and systematized, enabling GM to be more broadly understood and operationalized as a concrete instrument towards health equity.

## RESUMO

**Objetivos.:**

Analisar o progresso nas estruturas organizacionais, mecanismos, estratégias, e fatores habilitadores e barreiras para a integração do gênero (IG) na saúde na Guatemala, na Guiana e no Peru, dado o papel da IG na abordagem das desigualdades de gênero na saúde como um dos principais impulsionadores estruturais da equidade em saúde.

**Métodos.:**

Os dados foram obtidos por meio de uma análise das leis, políticas e/ou documentos de programa e entrevistas qualitativas semi-estruturadas junto a 37 informantes, extraídos da literatura cinza. A análise foi efetuada com base em uma estrutura teórica que incluiu 7 categorias consideradas essenciais para promover a IG no setor da saúde.

**Resultados.:**

Apesar dos esforços significativos e das experiências acumuladas na IG na saúde, as barreiras estruturais incluem: os desafios sociais mais amplos de transformar relacionamentos desiguais de poder; a complexidade dos sistemas de saúde associada à baixa capacidade técnica, política e financeira das instituições encarregadas pela IG; e a coordenação limitada (e muitas vezes fraca) com as Maquinarias Nacionais de Mulheres (NWM, na sigla em inglês). Em alguns contextos, as barreiras são exacerbadas pela compreensão limitada de conceitos básicos nas bases da IG (às vezes agravadas por mal entendidos relacionados à intersecção com homens e/ou o envolvimento deles); e a ausência de indicadores para medir resultados concretos da IG e seu impacto.

**Conclusões.:**

Para que a IG ocorra de fato, é necessária uma pauta mais estratégica e transformadora, elaborada e implementada em coordenação com as NWM e a sociedade civil, e fazendo referência a organismos externos (por exemplo, Comitê sobre a Eliminação de todas as Formas de Discriminação contra a Mulher) para que a integração inclua não só o processo, mas que forneça, além disso, uma diferenciação mais clara entre a sensibilidade às questões de gênero e a transformação das relações de gênero, e defina os resultados esperados e os indicadores para medir os avanços. A partir de então, elas poderiam ser melhor documentadas e sistematizadas, permitindo um melhor entendimento sobre a IG e sua operacionalização como instrumento concreto rumo à equidade na saúde.

Las desigualdades de género constituyen uno de los principales factores estructurales que impulsan la equidad en la salud, como señaló la Comisión sobre Equidad y Desigualdades en Salud en las Américas. Su informe prestó más atención que nunca a las “inequidades por razones de género” dentro de un marco conceptual que destacaba los factores estructurales políticos, sociales, culturales y económicos que impulsan las desigualdades y la necesidad de un enfoque interseccional que incluya la atención al género junto con las inequidades sociales y económicas, la sexualidad, el origen étnico, la discapacidad y la migración (1). Del mismo modo, abordar las desigualdades de género desde una perspectiva de equidad y derechos humanos es fundamental para el compromiso de que “nadie se quede atrás” de los Objetivos de Desarrollo Sostenible (ODS) (2).

Esta preocupación por la igualdad de género se refleja en la *Política de igualdad de género* de la Organización Panamericana de la Salud (OPS), adoptada en el 2005. También fue uno de los objetivos principales del proyecto de Sistemas Integrados de Salud en América Latina y el Caribe, un acuerdo de cooperación entre el Departamento de Asuntos Mundiales del Gobierno de Canadá y la OPS. Esta preocupación compartida impulsó el estudio que generó estos resultados, con el objetivo de investigar el grado en que, a nivel de país, la incorporación de la perspectiva de género en la salud está contribuyendo al logro de la igualdad de género y, por lo tanto, a determinar si puede ser necesaria una nueva visión y estrategia para la futura cooperación técnica de la OPS.

## Incorporación de la perspectiva de género para abordar las desigualdades de género y la equidad en la salud

A lo largo de las décadas transcurridas desde la adopción de la Plataforma de Acción de Beijing (3) y las conclusiones convenidas 1997/2 del Consejo Económico y Social de las Naciones Unidas (ECOSOC) (4), la incorporación de la perspectiva de género se ha convertido en el principal mecanismo destinado a lograr la igualdad de género y, por lo tanto, puede considerarse uno de los mecanismos clave para lograr la equidad en la salud (2). Esta incorporación pretende ser omnipresente para demostrar la pertinencia de las consideraciones de género en todos los aspectos de las políticas, los programas y los planes (incluso en la evaluación de sus consecuencias), y la salud pública no es una excepción. Su lógica inherente es que, sin enfoques diferenciados para tratar las dinámicas de género, es poco probable que las intervenciones de salud pública cumplan sus objetivos y que la acumulación de consideraciones de género en todas las acciones acabe por conseguir la igualdad de género en la salud. También subraya que la falta de esta perspectiva puede producir o perpetuar la desigualdad de género. Entre los ejemplos de incorporación de la perspectiva de género en la salud figuran, entre otros, la presentación de informes desglosados por sexo de los resultados de salud, el análisis de las diferencias en el acceso a los servicios entre hombres y mujeres, la inclusión de la mujer en la toma de decisiones y las respuestas adaptadas para satisfacer las distintas necesidades de hombres y mujeres, en particular mediante asignaciones presupuestarias específicas.

Si bien se considera que los mecanismos o instituciones nacionales de promoción de la mujer son las principales entidades responsables de la incorporación de la perspectiva de género, su función de coordinación denota una responsabilidad compartida (3) en todos los ámbitos políticos, incluida la salud.^1^ En consecuencia, las cuatro líneas estratégicas de la *Política de igualdad de género* de la OPS –desglose de los datos, fomento de capacidades, participación de la sociedad civil y seguimiento y evaluación– tratan de hacer realidad la incorporación de la perspectiva de género en los ministerios de salud.

## Marco conceptual

La incorporación de la perspectiva de género se basa en marcos teóricos feministas que reconocen las desigualdades profundamente arraigadas en las normas y estructuras sociales, con el objetivo de proporcionar una forma de abordarlas y lograr la transformación social. Por lo tanto, la incorporación de esta perspectiva nunca fue un fin sino una estrategia para alcanzar la igualdad de género. Su premisa operativa es que la formulación de políticas no es un proceso neutral en cuanto al género, sino que se basa en supuestos subyacentes con sesgo de género sobre cómo se reestructura y organiza la sociedad. Se centra en el proceso por el cual cualquier acción, legislación, política o programa se evalúa atendiendo a sus diferentes consecuencias en hombres y mujeres (4).

Aunque algunas críticas recientes han planteado que, en la práctica tecnocrática, la incorporación de la perspectiva de género se ha despolitizado y su potencial transformador previsto se ha debilitado (2), su conceptualización original requiere un cambio de paradigma en el diseño y la ejecución de políticas para determinar y abordar los supuestos con sesgo de género, y corregir la discriminación de género y las estructuras, los sistemas y las prácticas que se diseñaron inconscientemente con los hombres como modelo. Desde el punto de vista operativo, todas las acciones se orientarían, o reorientarían, con el fin de asegurar un impacto positivo en la igualdad de género en la salud.  La medida en que esto se logre depende del enfoque adoptado. La  incorporación de la perspectiva de género suele adoptar dos formas distintas en los extremos de un abanico de enfoques. En un extremo se sitúa la sensibilidad de género, que tiene en cuenta y promueve la concientización sobre las desigualdades de género y cómo afectan a cualquier acción, pero no pretende cambiarlas (5). En el otro extremo se encuentran los enfoques transformadores de género, cuyo objetivo es cambiar las relaciones de poder entre los géneros y transformar las normas, los roles y las relaciones de género perjudiciales (5).

La incorporación de la perspectiva de género se distingue por las medidas complementarias de acción positiva dedicadas específicamente a ocuparse de las prioridades y las necesidades de las mujeres y las niñas. Las medidas van desde los enfoques de la salud de la mujer que hacen hincapié en la salud reproductiva y la violencia contra la mujer hasta programas específicos dedicados a destacar el género como determinante estructural de la salud de las mujeres y los hombres, centrados en modificar las relaciones desiguales de poder y subordinación, así como en permitir el empoderamiento de la mujer para acceder a los recursos de salud (6). Más recientemente, las acciones encaminadas a la “integración de la diversidad” o los enfoques de interseccionalidad también tienen por objeto contribuir a la igualdad de género en la salud y, por ende, a la equidad en la salud.

## Objetivos del estudio

En el 2018, la OPS llevó a cabo el presente estudio en Guatemala, Guyana y Perú para examinar las experiencias de incorporación de la perspectiva de género en el sector de la salud con el fin de analizar cualitativamente los avances logrados en cuanto a las estructuras organizativas, los mecanismos y las estrategias, así como los factores facilitadores y los obstáculos clave hacia la institucionalización de esta perspectiva. Se preveía que este análisis contribuiría a comprender mejor si la propia estrategia de incorporación de la perspectiva de género se enfrenta a retos para cumplir sus objetivos, o si los factores contextuales de su aplicación son el factor clave de su éxito o fracaso. El estudio se basó en las conclusiones del informe regional complementario (6), en el que se analizaron las definiciones de la transversalización o integración de la perspectiva de género y los requisitos institucionales establecidos en los documentos de política y los mandatos regionales, y se realizó una revisión sistemática de los resultados documentados de la transversalización del género en las políticas y los programas nacionales de salud, los documentos de la OPS, las publicaciones científicas y la información disponibles en la web; y entrevistas semiestructuradas con expertos regionales. En sus recomendaciones se abogaba por un mayor número de programas de integración de la perspectiva de género basados en los resultados, junto con estrategias definidas para el empoderamiento de las mujeres y la necesidad de continuar con el fortalecimiento institucional. El presente estudio constituyó un examen más profundo de los avances hacia resultados transformadores o de otro tipo en materia de igualdad de género logrados a través de estos mecanismos, a partir de estudios de caso.

En las secciones siguientes se presentan algunos resultados destacados de este estudio, que se complementan con el análisis de más bibliografía gris para situarlos mejor en sus contextos institucionales y normativos.^2^

## MÉTODOS

En este estudio de varios casos se obtuvo información mediante métodos cualitativos: revisión de documentos de política emitidos desde el 2015 hasta la fecha y 37 entrevistas semiestructuradas (15 en Perú, 9 en Guyana y 13 en Guatemala) en las que participaron 46 informantes expertos en total. Estos representaban a las autoridades nacionales (ministerios de salud y otros ministerios encargados de supervisar el desarrollo, la inclusión social, la justicia, la mujer y las cuestiones de género), organizaciones de mujeres, académicos y organismos de las Naciones Unidas. Todas las entrevistas se grabaron con el consentimiento informado de los participantes y acuerdos de confidencialidad y anonimato.

**FIGURA 1.**
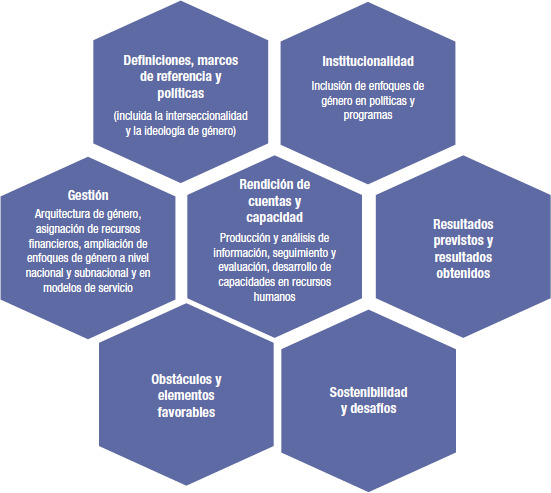
Siete categorías consideradas esenciales para avanzar en la integración de la perspectiva de género en el sector de la salud

El análisis es tanto descriptivo, con respecto a las experiencias de incorporación de la perspectiva de género en el sector de la salud, como explicativo, ya que los hallazgos se analizaron sobre la base de un marco teórico, incluidas siete categorías consideradas esenciales para el avance de esta perspectiva en el sector de la salud (figura 1). Las conclusiones que aquí se presentan proceden principalmente del análisis de los datos recogidos en las entrevistas, y la revisión de los documentos sirve de contexto a efectos informativos.

El estudio no fue una evaluación, un análisis de buenas prácticas ni un estudio representativo. La selección de los países se basó en la inclusión en el financiamiento del proyecto de Sistemas Integrados de Salud en América Latina y el Caribe, en la representación de las tres subregiones (América Central, América del Sur y el Caribe), en los países prioritarios del Plan Estratégico de la OPS 2014-2019, y en la aprobación del Gobierno. Aunque las conclusiones pretenden ser comparables en general, no se pueden establecer comparaciones directas entre los países. Debido al número limitado de participantes y a los problemas de identificación, el análisis no trató de exponer las diferentes perspectivas según cada sector (p. ej., organismos de las Naciones Unidas, autoridades nacionales, sociedad civil), aunque esta puede ser una línea de investigación útil en el futuro.

## RESULTADOS

Para que el debate fluya de forma concisa en este artículo, la presentación de conclusiones no se estructura según las categorías analíticas originales, sino más bien en función de lo siguiente: institucionalización; definiciones que orientan las acciones de incorporación de la perspectiva de género; diversidad de experiencias concretas en esta incorporación. Estas categorías recogen resultados de las siete categorías analíticas mencionadas anteriormente.

### Institucionalización

Dado que la integración de la perspectiva de género se centra en el proceso por el que se produce el cambio (4), se considera fundamental la arquitectura de género utilizada para impulsar el cambio transformador. En lo que respecta a su gestión y establecimiento de una agenda, se observa que la institucionalización de la incorporación de la perspectiva de género en los países es variada y se materializa en dos niveles: en de los ministerios de salud y en las instituciones nacionales de promoción de la mujer. Dentro del Ministerio de Salud, la institucionalización consiste en diversas instituciones y estructuras independientes de asesoramiento y coordinación, y en unidades o puntos focales de género. Estas suelen estar adscritas a las oficinas ministeriales y también pueden encontrarse a nivel subnacional. Los diferentes mecanismos pueden desempeñar un papel importante en la institucionalización sostenible de la incorporación de la perspectiva de género en la salud. Sin embargo, por lo general no están integradas estratégicamente en el aparato institucional y su estructura es débil, sus acciones dispersas y su capacidad de coordinación con los diferentes programas o divisiones de los ministerios es escasa.

Aunque las entidades con las que debe coordinarse el Ministerio de Salud difieren en cada contexto, las instituciones nacionales de promoción de la mujer tienen, en teoría, responsabilidades generales de supervisión, asesoramiento y coordinación de la incorporación de la perspectiva de género en todos los ámbitos políticos, incluida la salud. En la práctica, la consolidación institucional de los tres mecanismos nacionales varía considerablemente, lo que repercute en su capacidad para asesorar y apoyar eficazmente a las autoridades de salud. En Perú, el mecanismo nacional de promoción de la mujer ha alcanzado el mayor nivel de consolidación institucional, al convertirse en un ministerio de pleno derecho (7). Pese a ello, carece de los recursos adecuados para desempeñar su mandato, lo que limita su capacidad de establecer una agenda efectiva para la igualdad de género en materia de salud, en coordinación con el Ministerio de Salud.^3^ Esto es aplicable sobre todo a las necesidades de las mujeres y las niñas que se enfrentan a formas de discriminación, desigualdad y exclusión que se entrecruzan.^4^

En Guatemala, la Secretaría Presidencial de la Mujer (SEPREM) fue creada en el año 2000 como la entidad asesora encargada de coordinar las políticas públicas destinadas a impulsar la promoción de la mujer y la igualdad de género, teniendo presente la diversidad sociocultural del país. A pesar de no ser un ministerio propiamente dicho, la SEPREM siempre ha estado adscrita directamente al gabinete de la Presidencia y su titular tuvo rango ministerial hasta el 2016 (7). Se ha señalado regularmente la preocupación por la falta de coordinación efectiva de la SEPREM y la división poco clara de su labor con otras instituciones y estructuras, también en el 2017 por el Comité de la Convención sobre la Eliminación de Todas las Formas de Discriminación contra la Mujer (CEDAW, por su sigla en inglés) (11), algo que obstaculiza su función consultiva. Se han hecho llamamientos para aumentar la capacidad de la SEPREM reforzando su autoridad institucional, recursos humanos y recursos financieros sostenibles (11).

En Guyana, la oficina encargada de los asuntos relacionados con la igualdad de los sexos es un mecanismo nacional de la mujer de bajo rango adscrito al Ministerio de Protección Social. La adscripción administrativa de las instituciones nacionales de promoción de la mujer a los ministerios responsables de asuntos sociales, familia, infancia y afines es una característica común de los Estados Miembros de CARICOM (12) que transmite los papeles tradicionales de la mujer en la sociedad, tendencia que, por otra parte, ha sido ampliamente superada en América Latina (7). Esta posición de bajo perfil se traduce en una autoridad limitada y dificulta la coordinación que se requiere para que la incorporación de la perspectiva de género sea una realidad con las autoridades sectoriales, como el Ministerio de Salud, tal y como señaló el Comité de la CEDAW en el 2019 (13).

La complejidad de los diferentes arreglos institucionales en los países produce una sobrecarga en las “unidades” de cuestiones de género que se ven obligadas a actuar de forma proactiva y reactiva con una doble carga de coordinación: con las instituciones nacionales de promoción de la mujer y en los ministerios de salud. Esto hace que las tareas de coordinación sean más complejas y genera una posible confusión sobre dónde recae la responsabilidad de incorporar la perspectiva de género. Crea además dificultades a la hora de adoptar una estrategia coherente y contextualizada sobre la incorporación de la perspectiva de género en el ámbito de la salud, lo que se complica aún más por los intentos de aplicar un enfoque interseccional. En Guatemala, por ejemplo, la Unidad de Atención a la Salud de los Pueblos Indígenas e Interculturalidad tiene como objetivo definir estrategias para integrar las respuestas a las especificidades de las poblaciones indígenas. No obstante, funciona independientemente de la unidad de género, lo que impide adoptar un enfoque de género que tenga más en cuenta la equidad en lo que respecta a la diversidad de los distintos grupos de hombres y mujeres.

Es posible que Perú sea el país con la tendencia más clara hacia la incorporación de la perspectiva de género como objetivo político y marco normativo. En cambio, aunque se han realizado esfuerzos innovadores a través del mecanismo nacional de promoción de la mujer, los intentos registrados en Guatemala son más aislados. Predomina la cooperación sobre temas concretos, en lugar de una labor sistemática de integración más amplia en  el ámbito de la salud. Esto indica que la institucionalidad es relativamente débil dentro del Ministerio de Salud y que los obstáculos a los que se enfrenta el mecanismo nacional de promoción de la mujer son continuos. Los esfuerzos desplegados en Guyana están aún más fragmentados. El mecanismo nacional de promoción la mujer no ha desempeñado un papel central en la coordinación o el asesoramiento sobre la incorporación de la perspectiva de género en las políticas e intervenciones de salud, y las autoridades de salud tampoco han compensado esta debilidad con estructuras y recursos sólidos.

En todos los casos, la institucionalización dentro del sector de la salud constituye el flanco más débil en lo que respecta a la incorporación. Las iniciativas son frágiles (en términos de recursos humanos y financieros y de seguimiento), discontinuas (los cambios de mecanismo institucional o de estrategia son habituales) y específicas (sin un plan de acción). Este hecho se refleja en una fuerte concentración en las políticas, las leyes y los marcos regulatorios, pero en una incipiente aplicación dentro del sector de la salud.

Dejando a un lado los retos, el marco normativo legislativo y de políticas en materia de género ha avanzado, especialmente en Perú, seguido de Guatemala, con novedades más recientes en  Guyana. Los tres países han ratificado la CEDAW (aunque solo Perú y Guatemala han ratificado su Protocolo Facultativo) (14). Sin embargo, solo Perú ha promulgado una legislación específica en materia de igualdad de género (15), adoptando un Plan Nacional de Igualdad de Género 2012-2017 que posteriormente sirvió de base a la Política Nacional de Igualdad de Género destinada a abordar la discriminación estructural que sufren las mujeres (16). En Guatemala se ha adoptado una Política Nacional de Promoción y Desarrollo Integral de las Mujeres y un Plan de Equidad de Oportunidades 2008-2023 (17). En el 2019, una vez concluida la investigación, Guyana formuló la política nacional de igualdad de género e inclusión social como un marco que sirva de orientación en la implementación de mecanismos, políticas y protocolos adecuados para resolver las cuestiones de desigualdad de género y exclusión social (18).

### Definiciones que orientan las acciones de incorporación de la perspectiva de género

A pesar de estos retos, en los tres países se han llevado a cabo iniciativas encaminadas al objetivo de la igualdad de género, haciendo referencia al género en el sector de la salud y en otras áreas del Estado y existe, sin lugar a dudas, un conocimiento acumulado con respecto a la incorporación de la perspectiva de género. No obstante, este compromiso firmemente declarado oculta una diversidad de definiciones de conceptos manejados bajo el paraguas de la incorporación de la perspectiva de género.

Uno de los objetivos más aceptados es el de la “inclusión” de las voces y necesidades de las mujeres, los hombres y la población LGBTI. Ahora bien, la comprensión y la operacionalización de esa inclusión no son uniformes. Por ejemplo, las necesidades estratégicas de las mujeres relacionadas con la salud sexual y reproductiva y los derechos conexos tienen prioridad en Guatemala y Perú, pero no en Guyana. Todo ello a pesar de los desafíos existentes en Guyana, como la necesidad de reformas legislativas para acabar con el matrimonio precoz y forzado y la existencia de prácticas perjudiciales como la mutilación genital femenina, según lo señaló en el 2019 el Comité de la CEDAW (13). En cambio, la incorporación de la perspectiva de género en Guyana pone por delante la aplicación de acciones positivas para incluir explícitamente a los hombres en la elaboración de políticas, con el objetivo de representar mejor sus diferentes necesidades y, de hecho, trabajar con mujeres se considera algo “anticuado”. En Guatemala, la agenda de la “igualdad de género” también está cambiando con un enfoque incipiente en la diversidad sexual y la salud de las personas LGBTI.

La inclusión de estos temas emergentes no es intrínsecamente problemática, todo dependerá de la intención con que se orienten. En realidad, la inclusión de las cuestiones LGBTI resulta necesaria para cumplir con las exigencias de la CEDAW de hacer frente a formas de discriminación contrapuestas. Sin embargo, puede justificar la preocupación por la disminución de los recursos y la atención prestada al empoderamiento de las mujeres o representar un malentendido de los objetivos transformadores de la incorporación de la perspectiva de género (5), especialmente en el contexto de que la institucionalización sea frágil para orientar de forma adecuada las agendas.

### Diversidad de experiencias concretas en la incorporación de la perspectiva de género

En este marco se han realizado acciones concretas de incorporación de la perspectiva de género, ya sea como iniciativas nacionales o como acciones dispersas en niveles subnacionales o en otras instituciones fuera del Ministerio de Salud. En términos generales, se han puesto en marcha acciones dedicadas a temas determinados que se consideran necesidades estratégicas de las mujeres y que están vinculadas a los ODS, principalmente la salud sexual y reproductiva y los derechos conexos, la prevención de la violencia sexual y de género, la anticoncepción y la mortalidad materna, o como acciones fragmentadas y episódicas relacionadas con el proceso que se enmarcan en las dimensiones de la política de incorporación de la perspectiva de género de la OPS. Esto incluye reforzar la capacidad, crear mecanismos institucionales y fortalecer la recopilación de datos desglosados por sexo en los sistemas de información de los tres países y, además, elaborar presupuestos con un enfoque de género en Guatemala.

Los esfuerzos de fortalecimiento de la capacidad son notables, en particular la capacitación para el personal de salud y los servicios en áreas programáticas prioritarias, sobre todo en torno a la salud sexual y reproductiva y los derechos conexos, la violencia contra las mujeres o los programas de mortalidad materna. No obstante, estas iniciativas no suelen estar presentes en la capacitación más general e institucionalizada de los recursos humanos y los contenidos curriculares no parecen estar estandarizados.

Se han producido importantes avances en la inclusión de  variables, como el sexo, en los sistemas de información de salud. Estos logros a menudo se han visto acompañados de variables como la etnia o la identidad sexual, que son importantes para un análisis interseccional desde una perspectiva de equidad más amplia. Aun así, este tipo de análisis interseccional no es habitual y con frecuencia solo se produce en el caso de los perfiles de género y salud apoyados por la cooperación internacional. El seguimiento es el componente más frágil de la incorporación de la perspectiva de género. No se efectúa de forma sistemática o uniforme en los ministerios de salud o en las instituciones nacionales de promoción de la mujer. Además, no existen herramientas y prácticas de seguimiento para medir los avances hacia la igualdad de género en materia de salud. La excepción son las áreas para las que ya hay indicadores, como la mortalidad materna.

La asignación de recursos financieros destinados a la incorporación de la perspectiva de género es limitada en todos los países, si bien hay algunos mecanismos que permiten evaluar su magnitud. El esfuerzo más notorio es el clasificador presupuestario de Guatemala que registra y clasifica las asignaciones presupuestarias de los programas, subprogramas, proyectos y actividades orientados al “desarrollo integral de las mujeres guatemaltecas”.^5^

## DISCUSIÓN

En el presente estudio se determinó un conjunto de obstáculos y factores facilitadores importantes para avanzar en la incorporación de la perspectiva de género, varios de los cuales pueden caracterizarse como de naturaleza estructural, lo que quizá otorgue cierta importancia a la despolitización de la perspectiva de género en los esfuerzos de incorporación, debilitando así su objetivo transformador originalmente declarado (2, 21).

El mayor obstáculo estructural es la persistencia de sociedades que se enfrentan a retos más amplios de transformar la desigualdad en las relaciones de poder entre mujeres y hombres, así como la discriminación y la exclusión conexas. El sector de la salud no es ajeno a las normas y valores de la sociedad en general, y estos impregnan a los proveedores y los responsables de la toma de decisiones. La complejidad del sistema de salud, y su multiplicidad de actores, puede hacer que abordar la cultura de la desigualdad de género resulte aún más difícil que en otras instituciones sociales relevantes. Volviendo a recordar la posible desconexión entre las agendas feministas y la integración institucional (21) y quizás simplemente teniendo en cuenta las realidades de las agendas nacionales que compiten por recursos limitados, la capacidad de abordar realmente estas complejidades no se ve favorecida por la escasa capacidad técnica, política y financiera de las estructuras institucionales, a menudo excesivamente complejas, a las que se ha encomendado la responsabilidad de la incorporación de la perspectiva de género.

Por otra parte, cada vez se reconoce más la necesidad de la igualdad de género en la salud formulada, al menos en parte, como respuesta a las prioridades de la agenda feminista. Entre los aspectos favorables para impulsar la igualdad de género en la región se encuentran la existencia de normativas regionales y mundiales, y las capacidades a nivel nacional que se han incrementado directamente en respuesta a las mismas, así como a las prioridades establecidas por la sociedad civil a nivel nacional. Guatemala se beneficia de un legado de dos décadas de experiencia acumulada por varias estructuras e instituciones encargadas de velar por los derechos de las mujeres y la igualdad de género (7), que incluye una alianza del mecanismo nacional de promoción de la mujer con grupos de mujeres, incluidas las mujeres indígenas (7) (lo que demuestra un intento de fortalecer, en lugar de debilitar, la agenda sobre la igualdad de género a través de la interseccionalidad, incorporando las realidades de diferentes grupos de mujeres). Asimismo, el modelo de atención de salud recientemente aprobado sirve de plataforma para promover la incorporación de la perspectiva de género en el sector y el trabajo relacionado con el clasificador de género en el presupuesto está diseñado para mejorar la responsabilidad por los resultados.

Al igual que en Guatemala, el papel de la sociedad civil en Perú y Guyana también está contribuyendo a llamar la atención sobre cuestiones clave de la agenda feminista, como la violencia contra las mujeres y la necesidad de una mayor participación de la mujer en los puestos de decisión, de forma que se contrarreste la despolitización y estas cuestiones se integren en las labores relativas a la incorporación de la perspectiva de género. En el caso de Perú, por ejemplo, se destaca la formulación de una nueva versión de la política de género en materia de salud y su implementación. Del mismo modo, en Guyana, la formulación de la política nacional de igualdad de género e inclusión social (18) y, más concretamente en respuesta a la prioridad feminista de poner fin a la violencia contra las mujeres, la aplicación de la guía sobre violencia sexual dirigida al personal médico demuestra la capacidad de respuesta a las agendas transformadoras.

A pesar de estos avances, persisten importantes obstáculos. Hay una comprensión limitada de los conceptos básicos y de las expectativas que se derivan de la incorporación de la perspectiva de género, y que aumenta cuando se introduce la interseccionalidad o el compromiso con los hombres. Esto ilustra la falta de una teoría del cambio para lograr la igualdad de género y, de hecho, la poca claridad en cuanto al objetivo primordial de la incorporación de la perspectiva de género en términos de capacidad de respuesta a las prioridades feministas, como las destacadas por el Comité de la CEDAW. La incorporación de la perspectiva de género no se entiende en relación con el examen de las desigualdades de género en los determinantes de la salud, los resultados y el acceso a los recursos para la salud. Resulta fundamental incluir a los hombres para sensibilizarlos sobre la necesidad de abordar las dinámicas de poder y empoderar a las mujeres. Sin embargo, su inclusión debido a una preocupación errónea por la falta de atención a las necesidades de los hombres denota neutralidad más que la conveniencia de corregir las dinámicas de poder arraigadas en las relaciones entre mujeres y hombres, que han acallado sistemáticamente las necesidades y las voces de las mujeres.

Asimismo, el creciente interés por la diversidad sexual puede confundir los significados que se atribuyen a la incorporación de la perspectiva de género. Dada su estrecha relación con la heteronormatividad y el control de la sexualidad de las mujeres, hacer frente a la discriminación de la población LGBTI es esencial y transformador para la agenda de empoderamiento de las mujeres. Pero cuando, como ocurre en algunos contextos, se entiende que el género se refiere principalmente a las cuestiones LGBTI, los esfuerzos pueden descentralizarse y los recursos se diluyen en detrimento del empoderamiento de las mujeres. Esto ilustra los riesgos de que se produzca una cooptación en el paso de la incorporación de la perspectiva de género a la incorporación de la diversidad y los enfoques de interseccionalidad (19, 20) cuando la primera no está clara, a pesar de que resulta fundamental un enfoque de interseccionalidad para afrontar la diversidad (21, 22) y la equidad en la salud. La falta de instrumentos para medir los resultados concretos de la incorporación de la perspectiva de género, más que el proceso, y especialmente la ausencia de indicadores de género que superen la visión del programa y que se relacionen con la igualdad de género en la propia salud, contribuyen a esta confusión en torno al objetivo y la visión de la incorporación de la perspectiva de género.

## Conclusiones

No hay que subestimar las limitaciones en cuanto a sostenibilidad de las estrategias de incorporación de la perspectiva de género representadas por factores como la inestabilidad política o la falta de asignaciones presupuestarias institucionalizadas de peso. Igualmente, es evidente que se necesita un cambio cultural considerable para que la incorporación de la perspectiva de género se aplique a un ritmo más sostenible en el ámbito de la salud pública. Esto debe ser contextualizado dentro de un esfuerzo social más amplio para cambiar las normas de género y abordar las desigualdades. Otros factores tienen que ver con la complejidad de un sector de la salud con múltiples facetas que hace imprescindible trabajar con áreas tan diversas como comunicación, planificación, epidemiología o financiamiento, al mismo tiempo que se avanza en los distintos programas nacionales o áreas temáticas, y en el plano de la prevención y la atención.

La coordinación con las instituciones nacionales de promoción de la mujer, la sociedad civil y la vinculación a entidades externas, como el Comité de la CEDAW, son vitales para que en la incorporación de la perspectiva de género se priorice un programa estratégico de transformación, coherente con los esfuerzos sociales más amplios encaminados a cambiar las normas de género y otras iniciativas emprendidas con el fin de lograr la equidad en la salud. Esta coordinación dentro de una agenda estratégica más definida y basada en una teoría del cambio permitiría que la incorporación de la perspectiva de género vaya más allá de centrarse en el proceso, con una distinción más clara entre los enfoques sensibles al género y aquellos transformativos de las relaciones desiguales de género, en busca de una mejor definición de los resultados previstos. La introducción de indicadores definidos para medir los avances permitiría documentar y sistematizar los resultados obtenidos.^6^

Si se definen, supervisan y documentan de este modo, es probable que las acciones de incorporación de la perspectiva de género se comprendan mejor, se institucionalicen, se planifiquen, se pongan en marcha y se financien como políticas estructurales con potencial de transformación capaz de influir en las desigualdades en materia de salud, lo mismo que otras agendas de desarrollo. Si esto ocurriera, la incorporación de la perspectiva de género podría funcionar más eficazmente a todos los niveles con un efecto de filtración en el que los enfoques transformadores de género llegarían a las realidades de la vida de las personas a nivel de proveedores y territorios, y se entenderían más fácilmente como un instrumento concreto de equidad en la salud. También puede facilitar la puesta en marcha de un enfoque contextualizado e interseccional, que trascienda un planteamiento genérico y mecanicista, orientado a los procesos, y que desarrolle estrategias de “inmersión profunda” de la incorporación de la perspectiva de género en la salud dentro de un marco más amplio de equidad para las mujeres que viven en situación de vulnerabilidad. Esto último resulta crucial, ya que, ahora más que nunca en la era de la COVID-19, los enfoques de género no pueden permitirse ser superficiales. Deben enfrentarse de forma concreta a las profundas desigualdades de género que actualmente están en la raíz de muchas de las inequidades en materia de salud en la Región de las Américas.

## Declaración.

Las autoras son las únicas responsables de las opiniones expresadas en el manuscrito, que no necesariamente pueden reflejar la opinión o la política de la *RPSP/PAJPH* o la OPS.

## Footnotes

1 Se trata de las instituciones nacionales que supervisan las cuestiones de la mujer, la igualdad de género y otros temas relacionados, aunque se utilizan distintos términos para denominar estos mecanismos en cada contexto.

2 En el contexto de este estudio, se entiende por “bibliografía gris” las publicaciones (documentos oficiales, publicaciones e informes de investigación) de las instituciones y organismos de las Naciones Unidas, así como los documentos normativos y de política gubernamental, como se detalla en la lista de referencias.

3 Este hecho fue observado con preocupación por el Comité para la Eliminación de la Discriminación contra la Mujer (CEDAW) en el 2007 (8) y el 2014 (9), aunque en enero del 2020 (una vez concluida la investigación para este artículo) Perú informó, como parte de su presentación periódica ante el Comité de la CEDAW, que el presupuesto del Ministerio de la Mujer y Poblaciones Vulnerables se había duplicado entre el 2012 y el 2017, con un aumento medio de casi el 20% anual durante ese período (10).

4 El Comité de la CEDAW expresó su pesar por la falta de “información específica con respecto a las medidas dirigidas a abordar la discriminación y la violencia a las que se enfrentan determinados grupos desfavorecidos de mujeres (...) que afrontan formas múltiples e interrelacionadas de discriminación” y reiteró su preocupación “porque las mujeres rurales e indígenas en particular sigan enfrentándose a barreras en el ejercicio de sus derechos y en el acceso a los servicios básicos”, por ejemplo la salud y la falta de “instrumentos de política existentes para abordar sus especificidades” (9).

5 El clasificador tiene por objeto cuantificar los recursos asignados a las cuestiones de género y orientar el proceso de planeación, y se aplicó por primera vez en el 2018 en el sector de la salud mediante 7 programas y 166 subproductos, que correspondió a un monto aproximado de 600 millones de pesos guatemaltecos de un presupuesto total del Ministerio de Salud que ascendió a 13 000 millones.

6 Por ejemplo, la OPS ha propuesto “un marco conceptual e indicadores para monitorear la igualdad de género en la salud en las Américas”, que incluye un marco actualizado y un conjunto de indicadores básicos para dar seguimiento a los avances en la igualdad de género en la salud en la Región (23).
